# De Novo Assembly, Characterization and Comparative Transcriptome Analysis of the Mature Male and Female Gonads in *Acrossocheilus parallens*

**DOI:** 10.3390/ani15060806

**Published:** 2025-03-12

**Authors:** Weiqian Liang, Lanyuan Liu, Dingxian Chen, Kaifeng Wang, Shengyue Lin, Weijian Chen, Sixun Li, Binhua Deng, Qiang Li, Chong Han

**Affiliations:** School of Life Sciences, Guangzhou University, Guangzhou 510006, China; liangweiqian1112@126.com (W.L.); 13539040894@163.com (L.L.); cdx920021@163.com (D.C.); 13530626652@163.com (K.W.); 13923505936@163.com (S.L.); 13048201080@163.com (W.C.); 18718617213@163.com (S.L.); d18206646398@163.com (B.D.)

**Keywords:** *Acrossocheilus parallens*, transcriptome analysis, sex differentiation, gonad development, reproduction

## Abstract

*Acrossocheilus parallens* has become an important commercial aquaculture species in southern China due to its high nutritional content and ornamental value. However, at present, there is very little research on its gonad development and reproductive regulation, which has restricted the development of its aquaculture industry. In this research, the gonadal transcriptome sequencing data of female and male *A. parallens* were first analyzed and compared. A total of 67,251 unigenes were successfully assembled and a total of 34,069 unigenes were annotated. After the comparative transcriptome analysis, a sum of 14,514 differentially expressed genes (DEGs) were identified between the male and female gonads, with 9111 having significantly high expression in the testes and 5403 having high expression in the ovaries. Additionally, 82 DEGs related to reproduction, gonad development and differentiation in the gonads were identified and the differential expression profiles of partial genes were further validated using real-time fluorescence quantitative PCR. These results provide basic data for further research on the functions of the genes and pathways related to sex differentiation and gonad development in *A. parallens*.

## Simple Summary

*Acrossocheilus parallens* is a high-quality economic fish species with both ornamental and high nutritional value in southern China. However, there is still a gap in the research on the regulation of sex differentiation and gonad development in *A. parallens*. In this study, the gonad transcriptome analysis was first carried out using Illumina Novaseq technology. Through the transcriptome comparison of gonads between males and females, many differentially expressed genes related to steroidogenesis, gonad development and gametogenesis were identified. In addition, the expression levels of some typical reproduction-related genes in *A. parallens* were similar to other species. This study will provide valuable information for further research on the regulation of the reproduction of *A. parallens*.

## 1. Introduction

*Acrossocheilus parallens*, belonging to the subfamily Barbinae and order Cypriniformes, is mainly distributed in the Guangdong and Jiangxi provinces of China [1,2,3]. *A. parallens*, a kind of omnivorous stream fish, lives in streams with clear water and relatively fast currents [4] and mainly feeds on algae and zooplankton. It is of significant ecological and economic importance due to its unique habitat preferences and high-quality flesh [5]. In addition, its bright body color and coordinated swimming posture give it certain ornamental value [6]. However, despite its importance, the reproductive biology and genetic information of *A. parallens* are relatively understudied; only its complete mitochondrial genome has been studied so far [7,8], which poses challenges for its conservation and aquaculture development.

The sex determination and gonadal development process of fish are usually regulated by genetic and environmental factors [9], and their mechanisms are more complex compared to other higher vertebrates [10], showing great plasticity. The sex determination of fish is firstly determined by genetic factors [11], and many related genes and pathways have been uncovered, such as forkhead box protein L2 (*foxl2*) [12,13], anti-Müllerian hormone Y-linked (*amhy*) [14], double sex- and mab-3-related transcription factor (*dmrt*) [15,16], transcription factor Sox-9 (*sox9*) [17], anti-Müllerian hormone (*amh*) [18], transforming growth factor-β signaling pathway [19] and WNT signaling pathway [20]. The reproduction of fish can be regulated through the process of steroidogenesis, gonad development and gametogenesis in the aquaculture industry. Thus, it becomes particularly important to identify and regulate the genes and pathways involved in the processes above.

With the rapid development of next-generation sequencing (NGS) technology, transcriptome sequencing has been effectively used to provide gene expression profiles and regulatory mechanisms in specific tissues or organs. In addition, due to its low cost and high throughput, NGS-based transcriptome sequencing technology has been widely applied, and many reproduction-related genes have been identified in many fish species including *Siganus oramin* [21], *Spinibarbus hollandi* [22], *Coreoperca whiteheadi* [23], *Scortum barcoo* [24], *Rachycentron canadum* [25], *Silurus asotus* [26], *Scatophagus argus* [27], *Acipenser sinensis* [28] and so on. Based on these identified genes, the reproduction process of the above fish can be regulated and can be beneficial for the aquaculture industry. But so far, few reproduction-related genes have been studied in *A. parallens* and the gap in information on the gonadal transcriptome still exists.

In the present study, the Illumina-based transcriptome sequencing and de novo assembly of female and male gonads were carried out in *A. parallens*. After comparative transcriptome analysis, a large number of differentially expressed genes (DEGs) that participated in sex differentiation and gonadal development were identified and discussed following quantitative real-time PCR (qRT-PCR). Our results filled in the blanks in the gonadal transcriptome data for *A. parallens*, and may contribute greatly to further research on its sex differentiation and gonadal development.

## 2. Materials and Methods

### 2.1. Sample Collection

In our study, six two-year-old sexually mature *Acrossocheilus parallens* individuals (three females and three males) were obtained from an aquaculture farm in Shaoguan, Guangdong province in China. The fish were anesthetized with MS222 (Sigma, St. Louis, MO, USA). The gonads from each individual were excised and stored in liquid nitrogen within 30 s. All animal experiments were conducted according to the guidelines and approval of the Experimental Animal Ethics Committee of the Guangzhou University of China.

### 2.2. RNA Extraction and Library Construction

The total RNA was extracted using the RNA isolator Total RNA Extraction Reagent (Vazyme, Nanjing, China) according to the manufacturer’s instructions. The concentration and purity of the extracted RNA were examined using the Nanodrop 2000 (Thermo Scientific, Wilmington, DE, USA). Then, the integrity and quantity of the RNA were determined by an Agilent 4200 Bioanalyzer (Agilent Technologies, Santa Clara, CA, USA). The RNA with a qualified quality was entered into the library construction process. The *A. parallens* transcriptome libraries were constructed using NEBNext^®^ Ultra™ RNA Library Prep Kit for Illumina^®^ (NEB, Ipswich, MA, USA). The mRNAs of *A. parallens* were enriched using magnetic beads with Oligo (dT). In a high-temperature environment with metal ions, the RNA was fragmented, and the first cDNA chain was synthesized with random hexamers, followed by the addition of enzyme, buffer and dNTPs (dATP, dTTP, dGTP, dCTP) to synthesize the second chain of cDNA. The synthesized double-stranded cDNA was purified by magnetic beads and repaired, additional A was ligated to the tail end of the cDNA and the sequencing connector was connected. The sorted magnetic beads were used for fragment size sorting, the sorted fragments were enriched by PCR and finally were purified to obtain the final library.

### 2.3. Library Sequencing, De Novo Assembly and Annotation

The qualified libraries were sequenced using the Illumina Novaseq6000 (Illumina, Inc., San Diego, CA, USA) high-throughput sequencing platform with a sequencing strategy of PE150 (Pair-End 150). There were no less than 6 GB of sequencing data for each library.

To obtain high-quality assembly results for subsequent analysis, the raw reads were filtered. In this process, the low-quality and the splice sequences were discarded and the clean reads obtained by fastp (version 0.18.0) [29]. Then, the de novo assembly was carried out using Trinity software v2.15.1 [30]. The quality of the assembly sequence was evaluated using N50 values, sequence length and BUSCO (http://busco.ezlab.org/, accessed on 2 November 2024).

Three forward and reverse reading frames were used to predict the coding region of Unigene and altogether produced six kinds of coding protein sequences. After obtaining the coding protein sequences, the protein sequences were compared with the non-redundant protein sequences (Nr) (https://www.ncbi.nlm.nih.gov/, accessed on 6 December 2024) and the Uniprot protein (https://www.uniprot.org/, accessed on 12 January 2025) database. Finally, the coding mode with the maximum score was considered the final coding mode of the unigene.

In addition, based on homology searches by BLASTP, these unigenes were annotated against major public databases, including Nr, the Kyoto Encyclopedia of Genes and Genomes (KEGG, http://www.genome.jp/kegg, accessed on 18 December 2024), Swissprot, the Clusters of Eukaryotic Orthologous Group database (KOG, http://www.ncbi.nlm.nih.gov/COG/, accessed on 19 December 2024) and Gene Ontology (GO, http://geneontology.org, accessed on 4 January 2025).

### 2.4. Identification of Differentially Expressed Genes (DESs) and Enrichment Analysis

The clean reads of each sample were first mapped to the assembled transcripts using hisat2 v2.1.0 [31]. According to the alignment results, RNA-seq by expectation-maximization (RSEM) [32] was used to further calculate the transcripts per million (TPM), the fragments per kilobase of the exon model per million mapped fragments (FPKM), coverage and the other gene-expression-related values of each transcript. Then, the Python V3.6 program prepDE.py was applied to convert the results into a format that could be identified by edgeR [33,34]. The genes with a false discovery rate (FDR) parameter below 0.05 and an absolute fold change ≥ 2 (|log2FC| > 2) were considered DEGs. Finally, according to these DEGs, the significant GO terms and KEGG pathways (FDR < 0.05) were further enriched by the clusterProfiler program in the R package after Fisher’s exact test and the Benjamini correction.

### 2.5. Validation of DEGs Using Quantitative Real-Time PCR (qRT-PCR)

To validate the reliability of DEGs from RNA-seq, a total of 16 putative DEGs related to reproduction, gonad development and differentiation were selected for quantitative real-time PCR (qRT-PCR) analysis. Firstly, based on the sequences of these 16 DEGs and the reference gene β-actin, specific primers were designed by Primer Premier 5.0 (Table 1). Then, the cDNA templates were synthesized using the HiScript III RT SuperMix for qPCR (+gDNA wiper; Vazyme), and the qRT-PCR amplifications were performed using the SYBR Green qPCR Mix (GDSBIO, Guangzhou, China) on a Roche LightCycler 480 real-time PCR system (Roche, Basel, Swiss). The qRT-PCR reaction included an initial denaturation step at 95 °C for 3 min, followed by 40 cycles of 95 °C for 10 s, 60 °C for 20 s and 72 °C for 20 s, and a final extension at 72 °C for 5 min, ending with a dissociation curve process. Each gene was tested for three independent biological replicates and three technical replicates, and the expression of each gene was normalized using the β-actin by the comparative CT (2^−∆∆CT^) method [35] and was shown as mean ± standard error.

## 3. Results

### 3.1. Overview of Transcriptome Assembly Results

**Figure 1:**
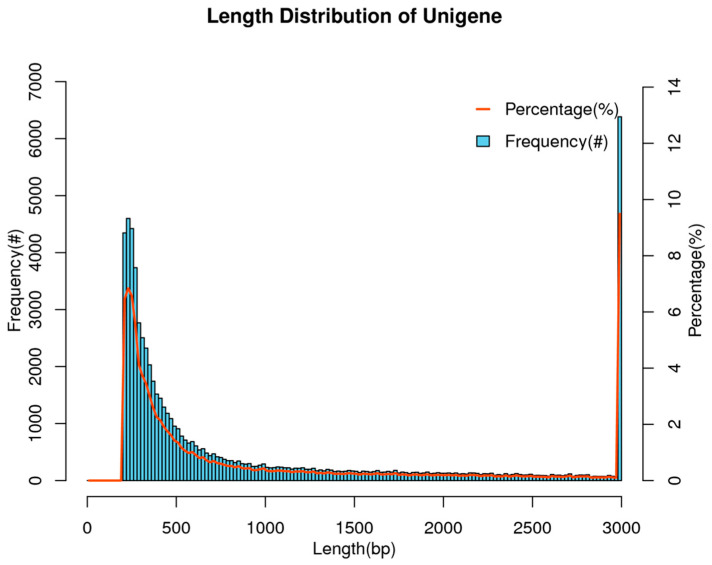
Length distribution of all the assembled unigenes of the *A. parallens* gonadal transcriptome. *X*-axis: length of unigenes; and *Y*-axis: frequency and percentage of unigenes at corresponding lengths.

A sum of six cDNA libraries, including three ovaries and three testes, was constructed by RNA-seq. After quality control and data filtering, a total of 35.71 GB clean reads were generated by using the Illumina HiSeq platform, with a mean of 5.95 GB and ranging from 5.57 to 6.17 GB per sample. The mean values of Q20 and Q30 were 97.26% and 93.06% (Table 2). After de novo assembly, a total of 67,251 unigenes were assembled, with the total length of these unigenes being 73,634,351 bp. The sequence length distribution of the unigenes ranged from 201 to 18,169 bp, with an average length and N50 of 1094 bp and 2416 bp, respectively (Table 3). Based on the predicted length statistics of the unigenes, most of the unigenes were 1–500 bp in length, and only a small number of them were 1000–3000 bp in length (Figure 1).

### 3.2. Unigene Annotation

**Figure 2:**
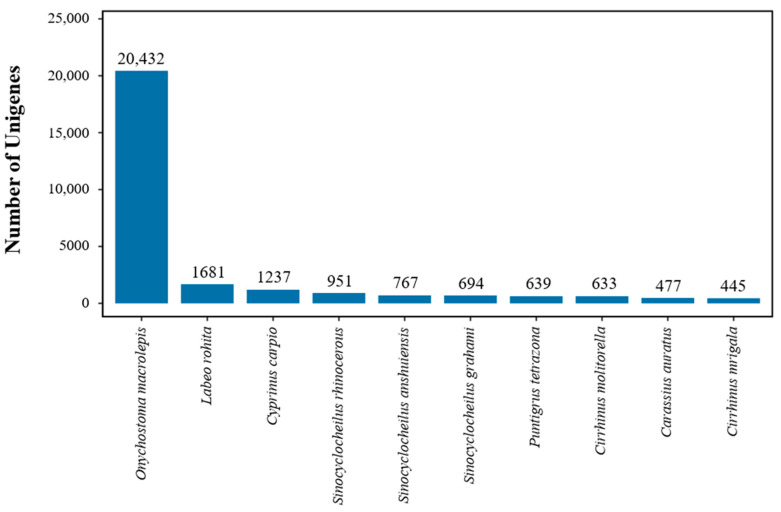
The species distribution of the results of Nr annotation. *X*-axis: the top 10 species which match the annotated sequence distribution; and *Y*-axis: the number of annotated sequences matching each species.

After annotation, a total of 34,069 unigenes were successfully annotated. Among all the annotated unigenes, 33,686 (98.88%) unigenes were matched with the Nr database, which had the highest percentage of annotated unigenes. The second highest percentage of annotated unigenes was in the KEGG database (32,251; 94.66%). However, there were only 18,137 (53.24%) unigenes compared to the KOG database, which may be related to the limited genomic information available for *A. parallens* (Table 3). Species distribution statistics were performed according to the most similar genes compared to the Nr database. The species with the largest number of genes indicated that the species contained the largest number of genes in the Nr database and was relatively similar to that of *A. parallens*. The results showed that *Onychostoma macrolepis* (20,432; 60.65%) had the biggest number of homologous genes to *A. parallens*, followed by *Labeo rohita* (1681; 12.84%), *Cyprinus carpio* (1681; 4.99%) and *Sinocyclocheilus rhinocerous* (1237; 3.67%) (Figure 2).

**Figure 3:**
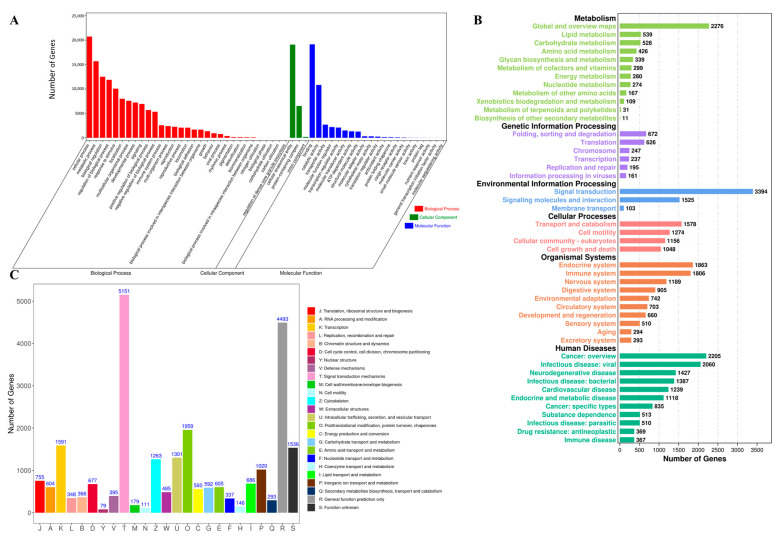
Functional annotation of unigenes based on GO (**A**), KEGG (**B**) and KOG (**C**) databases.

Additionally, all unigenes were further annotated in the GO, KOG and KEGG databases for their functional prediction and classification. In the GO database, a total of 23,051 (67.65%) unigenes were annotated and classified into three functional categories. Among these three functional groups, the terms “cellular anatomical entity” (98.88%), “binding” (81.32%) and “cellular process” (93.69%) were predominant in the cellular component, molecular function and biological process aspects, respectively (Figure 3A). Based on the KEGG database, the sum of 22,479 unigenes was divided into five different functional categories. The top three distributions were “Signal transduction” (3394 unigenes), “Global and overview maps” (2276 unigenes) and “Cancer: overview” (2205 unigenes) (Figure 3B). According to the KOG annotation, a total of 18,137 unigenes were grouped into 25 families. The richest distribution was “Signal transduction mechanisms” (5151 unigenes) followed by “General function prediction only” (4493 unigenes). The smallest distribution was “Nuclear structure”, with only 79 unigenes (Figure 3C).

### 3.3. Differential Gene Expression Analysis

**Figure 4:**
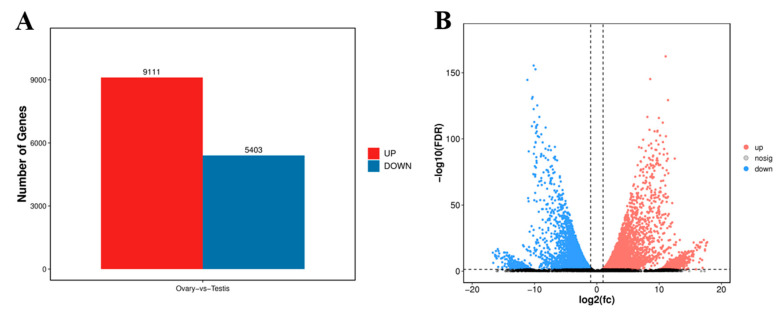
(**A**) DEGs in females (ovary) and males (testis) of *A. parallens*. (**B**) Volcano plot of DEGs in ovaries versus testes. Red and blue key dots, respectively, represent upregulated and downregulated genes in females, and vice versa.

**Figure 5:**
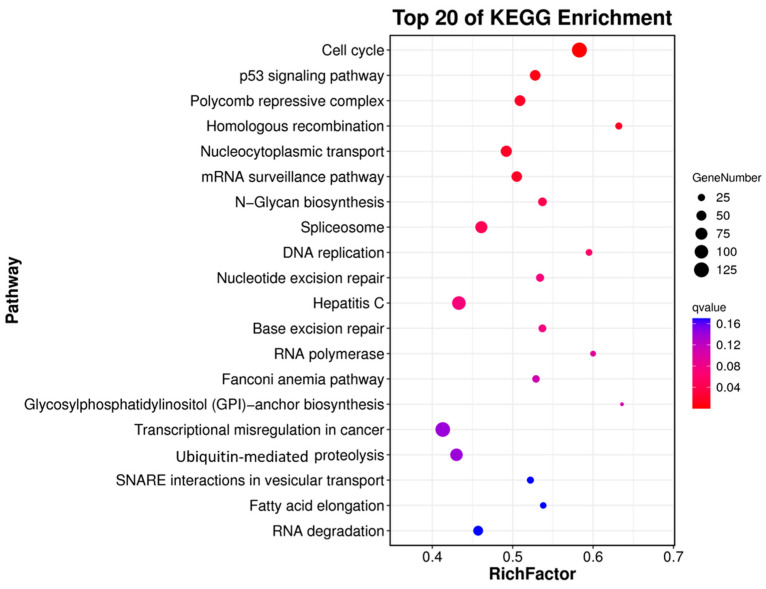
Top 20 pathways from KEGG enrichment analysis. *X*-axis: the ratio of the number of differential genes annotated to the KEGG pathway to the total number of differential genes; and *Y*-axis: the names of the enriched KEGG pathways. The size of the dots represents the number of genes annotated on the KEGG pathway term. The color of the dots represents the significant enrichment from red to blue.

The levels of gene expression were normalized by using the TPM values. A sum of 14,514 DEGs were identified between the ovary and testis samples, with 9111 (62.77%) DEGs being significantly highly expressed in the testes and 5403 (37.23%) being highly expressed in the ovaries (Figure 4A). The DEGs’ information is shown in the volcano plot (Figure 4). The enrichment analysis of these DEGs was further performed using GO and KEGG pathway analyses. The results of the KEGG enrichment analysis revealed that the cell cycle was the most representative, followed by the p53 signaling pathway and the polycomb repressive complex (Figure 5).

Moreover, based on the GO and KEGG annotation, numerous genes related to reproduction and gonad development and differentiation were identified (Table 4). Genes such as forkhead box protein J1-A (*Foxj1a*), double sex- and mab-3-related transcription factor 1 (*dmrt1*), protein Wnt-5a (*wnt5a*) and 11-beta-hydroxysteroid dehydrogenase type 2 (*hsd11b2*) were highly expressed in the testes. Meanwhile, bone morphogenetic protein 15 (*bmp15*), growth/differentiation factor 9 (*gdf9*), insulin-like growth factor 2a (*igf2a*), transcription factor SOX-11b (*sox11b*) and cytochrome P450 aromatase (*cyp19a1*) were highly expressed in the ovaries.

### 3.4. Validation of Transcriptomic Data by qRT-PCR

**Figure 6:**
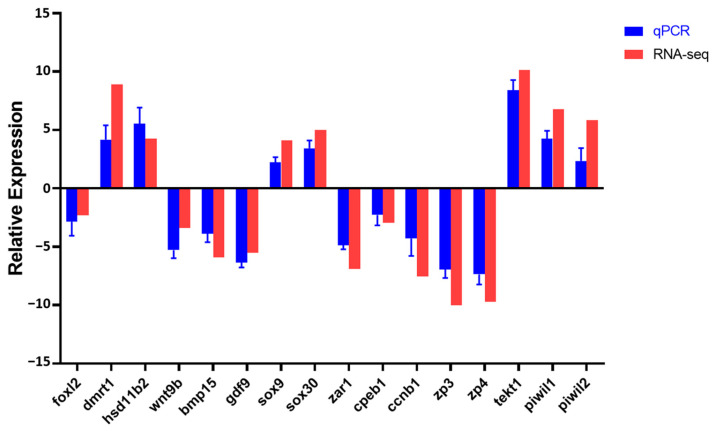
Validation of gonad transcriptome data by qRT-PCR.

A total of sixteen DEGs related to sex differentiation and gonadal development were analyzed from the transcriptome data, including *foxl2*, *dmrt1*, *hsd11b2*, *wnt9b*, *bmp15*, *gdf9*, *sox9*, *sox30*, *zar1*, *cpeb1*, *ccnb1*, *zp3*, *zp4*, *tekt1*, *piwil1* and *piwil2*. As expected, the expression patterns of qRT-PCR were consistent with the RNA-seq results (Figure 6).

## 4. Discussion

Sex differentiation is an extremely complex biological process, which contains a set of functional genes relating to gonad development, differentiation and maturity. Transcriptome has been proven in many species to be an effective sequencing method to obtain the overall information of the gene expression of specific tissues and to figure out the regulatory mechanisms. *A. parallens* is a high-quality commercial aquaculture species which has high nutritional and ornamental value. However, the gene expression and molecular mechanism during its sex differentiation process have not been revealed yet. Therefore, the comparative transcriptomic analysis of the ovary and testis was first conducted to confirm the DEGs related to sex differentiation in *A. parallens*.

### 4.1. DEGs Involved in Steroidogenesis Pathway

Sex steroid hormone, mainly referring to estrogen and androgen, plays an important role in regulating the sex differentiation in fish, and its concentration levels can affect the reproduction of fish. The hormones 17β-Estradiol (E2) and 11-ketotestosterone (11-KT), as the major endogenous estrogen and androgen in fish, respectively, were synthesized via the steroidogenesis pathway and were involved in the regulation of the gonadal sex differentiation in fish. A series of steroidogenic enzymes are required during the biosynthesis process of the sex steroid hormone and are encoded by a lot of genes, like *cyp19a1*, *foxl2*, *dmrt1* and *hsd11b2*.

Estrogen plays an important role in the regulation of sex differentiation and the maintenance of the female phenotype [36]. *Cyp19a1* encodes the aromatase catalyzing the conversion of androgen to estrogen as the terminal enzyme gene in the steroidogenic pathway in gonads. The inhibition of aromatase induced the sex reversal in female fish by blocking the synthesis of estrogen [37,38]. There have been studies suggesting that *foxl2* is involved in the regulation of *cyp19a1* promoters, and is used along with *cyp19a1* as the earliest known marker genes for ovarian sex differentiation [39,40]. For the sex differentiation in male fish, *dmrt1* is thought to be a crucial gene related to male sex differentiation, and showed antagonistic action against *foxl2* and decreased the transcriptional activity of *cyp19a1*, reducing the concentration levels of E2 in fish [16,41,42]. In addition, the biosynthesis of 11-KT from testosterone regulated by *hsd11b2* also affected the male sex differentiation from the perspective of synthesizing the androgen. The transcriptional activity of *hsd11b2* significantly increased during the spermatogenesis process, then maintained the development of the testis [43]. In *A. parallens*, the transcriptional levels of the *dmrt1* and *hsd11b2* genes were higher in the testis, while the transcription of *cyp19a1* and *foxl2* was more active in the ovary, suggesting that the gonad gene expression pattern on the steroidogenesis pathway was similar between different fish species [44,45,46]. Therefore, the results above indicated that these DEGs play crucial roles in gonad sex differentiation in fish.

### 4.2. DEGs Involved in Gonad Differentiation and Development

The development and differentiation of gonads are connected closely with sex differentiation in fish. Thus, it is essential to figure out the gene and corresponding mechanism involved in the process of gonad differentiation and development. Although the molecular mechanism of sex determination in fish is complicated, some members of gene families have been confirmed to regulate the development and differentiation of gonads, such as the *Wnt* gene family, *Bmp* gene family, the *Gdf* gene family and the *Sox* gene family [47,48,49].

The *wnt9b* expressed in the follicle is necessary for female sexual development. In zebrafish, the knockout of the *wnt9b* caused the female ovary to fail to develop properly and then to reverse into male testis [50]. In addition, *bmp15*, which fulfills a pivotal function in follicular development in mammals, similarly assumes an important role in the development of gonads in fish [51,52]. *Bmp15* has been demonstrated to be required for the maintenance of the female sex in juvenile zebrafish and cooperates with *gdf9* in the oocytes regulating the development and maturity of gonads on the Smad signaling pathway [53,54,55]. For male sex differentiation, *sox9* showed a testis-specific expression regulated by *dmrt1* and was then involved in testis development in Nile tilapia, which is consistent with its function in mammals [56,57]. The protein of *sox30* was detected abundantly in the spermatocytes and spermatid/sperm of carp testis. The transient knockdown of *sox30* resulted in the decrease in 11-KT and Hsd11b enzyme activity and downregulation of steroidogenesis-related genes like *hsd11b*, which then hindered the development of the testis [58]. In *A. parallens*, the transcriptional expression of *wnt9b*, *bmp15*, *gdf9*, *sox9* and *sox30* were, respectively, similar to the ovary and testis of many teleosts [59,60,61], which suggested that there was a considerable degree of similarity in the sex determination mechanism of the gonad in teleosts.

### 4.3. DEGs Involved in Gametogenesis and Gamete Maturation

Obtaining high-quality mature gametes is the ultimate but crucial step in the process of aquaculture practice. The regulation of gametogenesis and gamete maturation is associated with many gonadal genes such as *tekt1*, *piwil1*, *piwil2*, *zar1*, *cpeb1*, *ccnb1*, *zp3* and *zp4*.

*Tekt1* is a member of the *Tekt* gene family and encodes the protein that is involved in the formation of sperm flagella and is possibly related to flagellar stability and sperm motility [62]. The expression of *tekt1* in the testes was significantly decreased in the sterile triploid fish compared to the fertile tetraploid and diploid fish [63]. So far, many studies have demonstrated that *piwil1* and *piwil2* are highly conserved and essential in germ cell differentiation and sperm development in fish [64,65]. In *Paralichthys olivaceus*, *piwil1* and *piwil2* were expressed at higher levels in males and pseudo-males than in females, and they showed a significant positive correlation, indicating that they may play a coordinated function together in testes [66]. *zar1* is a conservative gene specifically expressed in the female gonads, playing essential roles during the oocyte-to-embryo transition [67]. A previous study has shown that the lack of *zar1* in tilapia led to arrested oogenesis with a significant decline in the germ cell number [68]. In addition, this study also indicated that *zar1* could interact with *cpeb1*, a component of the cytoplasmic polyadenylation machinery [69,70], to maintain early oogenesis [68]. As an important gene in the oocyte meiosis pathway, *ccnb1* has essential functions in the meiotic division of oocytes and in maintaining fertility [71], and it is highly expressed in the ovary, showing female-biased characteristics in zebrafish [72]. Furthermore, *zp3* and *zp4*, as important members of the sperm-binding protein gene family, are involved in regulating the reproduction process, forming the extracellular matrix around the oocyte and mediating sperm binding [73,74]. In *A. parallens*, the expression of *zar1*, *cpeb1*, *ccnb1*, *zp3* and *zp4* was higher in the ovary, while *tekt1*, *piwil1* and *piwil2* were higher in the testis. These genes were also found in *Spinibarbus hollandi* [22], *Scortum barcoo* [24] and *Siganus oramin* [21] and had similar expression levels and functions, indicating that the sex-biased genes related to gametogenesis and gamete maturation had extremely high conservation among teleost fish.

## 5. Conclusions

We carried out the de novo assembly of the gonads of *A. parallens* based on the Illumina sequencing technology, and a total of 67,251 unigenes were successfully assembled. The large number of DEGs that participate in sex differentiation and gonadal development were identified throughout the comparative transcriptome analysis between males and females, which have similar expression profiles with many teleost fish, indicating that they play conserved roles in the sex differentiation and gonad development process. Finally, the results of qRT-PCR confirmed that the expression pattern of these DEGs were reliable. The results of this study may provide valuable information for further research on sex differentiation and gonad development in teleost fish.

## Figures and Tables

**Table 1:** Primers used for qRT-PCR.

Gene	Sequence (5′–3′)	
	Forward Primer	Reverse Primer
*β-actin*	GTGTTGGCATACAGGTCCTTACG	ACGGACAGGTCATCACCATTG
*foxl2*	AGGGTTGGCAGAACAGTATCAGG	GAAATGCGTCGGTGGAGGTC
*dmrt1*	AACCCAAAGCAGCAGTTTTCTC	CGACAGAGAAGGTTCCCGAC
*hsd11b2*	AGACAGGCTAAAGGCCGGA	TGACGAAGTGTGTTGGTAAGAAGAT
*wnt9b*	CTCTGAGGGAATCTGTCCGC	GCGGTCTCTTTAAAGCCTCG
*bmp15*	TCCCAACCTCAAGTGACCTTC	ACGTGACTCTTGCCTCACAG
*gdf9*	CGAGCAAAACCGAGAGTTCTT	ATAGCAGAGCGATGTGAAGGG
*sox9*	AGGTCAGAGCTCCGGCTTGTACT	TGTGATTGGGTTGGGGAATGG
*sox30*	CCTTCTGGAGCAGAAAGTGAG	GTTGCTAGCATTAGGGTTGGC
*zar1*	CGTGAAGGAACCGCTTAGTC	TCTGCTCCAAAAACTGGAACC
*cpeb1*	CCTGGGACATCACTGAAGCTGG	TTGGGCATATTACCTTTCGGAGG
*ccnb1*	TTTACAACAGCTCGAGGTTGCG	GCATGAGCCAGTCGATGGTG
*zp3*	GCCTTCAGGTTCCACCAGGAC	GCCACCCATCTGTTGGTTTCG
*zp4*	TTGAGTGGTTGCTGGTGGTCC	CAGGTGTTTAGCGCTTTGTGG
*tekt1*	GGACAAATTTCAGGCCGAGC	AGGAGTCACTGCAGATCCAAG
*piwil1*	GCCATGTCTGAAGAAGCGATG	ATTAGCTGGCGAGTGTGGAC
*piwil2*	GCATCGTGACACTTTGCATTG	ACCCTGATCTTCTGCTGACTG

**Table 2:** Summary statistics of the gonadal RNA-seq data.

Sample	Number of Reads	Total Base	GC Content (%)	Q20 (%)	Q30 (%)
Ovary-1	41,216,910	6,083,067,389	45.86	97.02	92.49
Ovary-2	41,264,316	6,077,643,211	46.57	97.04	92.61
Ovary-3	40,229,586	5,882,642,501	45.89	96.92	92.42
Testis-1	41,869,770	6,171,999,520	45.77	97.14	92.75
Testis-2	37,750,646	5,572,829,473	48.31	97.34	93.15
Testis-3	40,272,500	5,922,275,444	47.78	98.12	94.94
Mean	40,433,955	5,951,742,923	46.70	97.26	93.06
Total	242,603,728	35,710,457,538			

**Table 3:** Summary statistics of the *A. parallens* gonadal transcriptome assembly and annotation.

Database	Number
Assembly	
Gene number (#)	67,251
Total length (nt)	73,634,351
Average length (nt)	1094
Max length (nt)	18,169
Min length (nt)	201
N50 (nt)	2416
GC	44.19%
Annotation	
Total number of annotated unigenes	34,069
Unigenes matched against Nr	33,686
Unigenes matched against UniProt	23,221
Unigenes matched against KEGG	32,251
Unigenes matched against KOG	18,137

**Table 4:** The patterns of DEGs related to reproduction, gonad development and differentiation in the gonads of *A. parallens* (Ovary_vs_Testis).

Unigene ID	log2FC	*p*-Value	FDR	Nr Annotation	Gene Name
Unigene0065503	17.164	3.71 × 10^−26^	2.59 × 10^−24^	tektin-4	*tekt4*
Unigene0020072	11.090	2.55 × 10^−3^	1.39 × 10^−2^	forkhead box protein G1	*foxg1*
Unigene0020990	11.048	5.63 × 10^−4^	3.58 × 10^−3^	paired box protein Pax-3a isoform X3	*pax3-a*
Unigene0039747	10.974	9.04 × 10^−3^	4.19 × 10^−2^	paired box protein Pax-2-A isoform X8	*pax2a*
Unigene0068389	10.156	1.21 × 10^−106^	2.88 × 10^−103^	tektin-1-like	*tekt1*
Unigene0063623	10.008	7.00 × 10^−15^	2.09 × 10^−13^	cytochrome P450 11B, mitochondrial	*cyp11b*
Unigene0012670	9.514	2.01 × 10^−84^	1.49 × 10^−81^	forkhead box protein J1-A	*foxj1a*
Unigene0031953	8.892	7.47 × 10^−77^	4.06 × 10^−74^	Double sex- and mab-3-related transcription factor 1	*dmrt1*
Unigene0042464	8.568	3.09 × 10^−10^	5.55 × 10^−9^	fibroblast growth factor 13b isoform X1	*fgf13*
Unigene0015615	8.523	3.27 × 10^−9^	5.11 × 10^−8^	bone morphogenetic protein 8A-like	*bmp8a*
Unigene0010324	8.393	8.15 × 10^−82^	5.29 × 10^−79^	sperm-associated antigen 16 protein	*spag16*
Unigene0039593	8.384	2.76 × 10^−89^	2.64 × 10^−86^	sperm-associated antigen 17 isoform X4	*spag17*
Unigene0043996	7.447	1.37 × 10^−3^	7.97 × 10^−3^	cytochrome P450 2K6-like	*cyp2k6*
Unigene0001198	6.693	1.01 × 10^−15^	3.25 × 10^−14^	protein Wnt-5a	*wnt5a*
Unigene0015091	6.776	1.25 × 10^−97^	1.90 × 10^−94^	piwi-like protein 1	*piwil1*
Unigene0001625	6.622	5.61 × 10^−17^	2.03 × 10^−15^	doublesex- and mab-3-related transcription factor A1-like	*dmrta2*
Unigene0033990	6.423	3.45 × 10^−5^	2.84 × 10^−4^	forkhead box protein P3 isoform X2	*foxp3*
Unigene0040562	6.174	1.60 × 10^−28^	1.29 × 10^−26^	protein Wnt-7b isoform X1	*wnt7b*
Unigene0005830	5.838	4.69 × 10^−48^	9.45 × 10^−46^	piwi-like protein 2	*piwil2*
Unigene0009058	5.561	4.38 × 10^−6^	4.25 × 10^−5^	fibroblast growth factor 14	*fgf14*
Unigene0039956	5.468	5.70 × 10^−12^	1.26 × 10^−10^	paired box protein Pax-8 isoform X1	*pax8*
Unigene0052496	5.011	1.43 × 10^−45^	2.58 × 10^−43^	transcription factor SOX-30-like isoform X2	*sox30*
Unigene0071169	4.442	1.22 × 10^−3^	7.19 × 10^−3^	transcription factor Sox-14	*sox14*
Unigene0017663	4.373	1.39 × 10^−4^	1.01 × 10^−3^	paired box protein Pax-3b isoform X1	*pax3a*
Unigene0064185	4.236	1.50 × 10^−22^	8.30 × 10^−21^	11-beta-hydroxysteroid dehydrogenase type 2	*hsd11b2*
Unigene0001099	4.191	1.68 × 10^−13^	4.37 × 10^−12^	insulin-like growth factor-binding protein 3	*igfbp3*
Unigene0014008	4.190	1.12 × 10^−5^	1.01 × 10^−4^	bone morphogenetic protein 3	*bmp3*
Unigene0071287	4.147	2.06 × 10^−26^	1.46 × 10^−24^	mothers against decapentaplegic homolog 5	*smad5*
Unigene0012314	4.110	7.88 × 10^−13^	1.91 × 10^−11^	transcription factor Sox-9-like	*sox9*
Unigene0030575	4.093	4.18 × 10^−11^	8.35 × 10^−10^	stAR-related lipid transfer protein 4 isoform X1	*stard4*
Unigene0063224	4.029	1.42 × 10^−16^	4.95 × 10^−15^	steroid hormone receptor ERR2 isoform X2	*esrrb*
Unigene0044603	3.879	9.20 × 10^−3^	4.26 × 10^−2^	fibroblast growth factor receptor 3 isoform X3	*fgfr3*
Unigene0066364	3.518	6.33 × 10^−9^	9.56 × 10^−8^	cytochrome P450 4B1	*cyp4b1*
Unigene0040138	3.446	4.82 × 10^−22^	2.55 × 10^−20^	forkhead box protein M1 isoform X2	*foxm1*
Unigene0035507	3.068	1.51 × 10^−4^	1.09 × 10^−3^	forkhead box protein L1	*foxl1*
Unigene0018532	2.972	9.64 × 10^−11^	1.85 × 10^−9^	growth/differentiation factor 10	*gdf10*
Unigene0069601	2.704	2.64 × 10^−6^	2.66 × 10^−5^	fibroblast growth factor receptor 2	*fgfr2*
Unigene0002103	2.687	3.68 × 10^−10^	6.52 × 10^−9^	stAR-related lipid transfer protein 9 isoform X1	*stard9*
Unigene0010222	2.671	1.04 × 10^−6^	1.12 × 10^−5^	fibroblast growth factor receptor-like 1	*fgfrl1*
Unigene0016545	2.461	4.25 × 10^−5^	3.43 × 10^−4^	cytochrome P450 4F3	*cyp4f3*
Unigene0040707	2.413	5.64 × 10^−3^	2.78 × 10^−2^	steroidogenic acute regulatory protein, mitochondrial	*star*
Unigene0057715	2.304	1.13 × 10^−4^	8.41 × 10^−4^	mothers against decapentaplegic homolog 4 isoform X2	*smad4*
Unigene0015555	2.257	4.05 × 10^−9^	6.25 × 10^−8^	forkhead box protein O4	*foxo4*
Unigene0058422	2.240	8.76 × 10^−6^	8.06 × 10^−5^	forkhead box protein J2	*foxj2*
Unigene0001059	2.139	4.00 × 10^−4^	2.64 × 10^−3^	estrogen-related receptor gamma a isoform X1	*esrrg*
Unigene0065036	2.127	5.45 × 10^−3^	2.69 × 10^−2^	cytochrome P450 7A1	*cyp7a1*
Unigene0049698	−1.662	7.54 × 10^−3^	3.59 × 10^−3^	protein Wnt-11	*wnt11*
Unigene0063444	−2.332	2.18 × 10^−5^	1.87 × 10^−4^	forkhead box protein L2a isoform X1	*foxl2*
Unigene0002571	−2.346	1.62 × 10^−6^	1.69 × 10^−5^	transcription factor SOX-4b	*sox4*
Unigene0004856	−2.461	2.53 × 10^−12^	5.81 × 10^−11^	very-long-chain 3-oxoacyl-CoA reductase-B	*hsd17b12b*
Unigene0001279	−2.512	9.74 × 10^−6^	8.89 × 10^−5^	androgen receptor isoform X2	*ar*
Unigene0016850	−2.522	4.59 × 10^−13^	1.14 × 10^−11^	forkhead box protein O3a	*foxo3a*
Unigene0058062	−2.648	1.48 × 10^−8^	2.13 × 10^−7^	mothers against decapentaplegic homolog 6a	*smad6a*
Unigene0020261	−2.913	1.21 × 10^−12^	2.88 × 10^−11^	3 beta-hydroxysteroid dehydrogenase type 7	*hsd3b7*
Unigene0064533	−2.940	8.16 × 10^−17^	2.92 × 10^−15^	cytoplasmic polyadenylation element-binding protein 1 isoform X1	*cpeb1*
Unigene0005781	−3.083	1.81 × 10^−17^	6.85 × 10^−16^	cytochrome P450 2J4	*cyp2j4*
Unigene0063752	−3.147	3.80 × 10^−21^	1.91 × 10^−19^	fibroblast growth factor receptor 1-A isoform X2	*fgfr1a*
Unigene0057571	−3.391	1.76 × 10^−8^	2.50 × 10^−7^	protein Wnt-9b	*wnt9b*
Unigene0008152	−3.417	3.87 × 10^−7^	4.46 × 10^−6^	paired box protein Pax-1a	*pax1*
Unigene0058533	−3.553	1.26 × 10^−14^	3.69 × 10^−13^	cytochrome P450 2F2-like isoform X1	*cyp2f2*
Unigene0021056	−3.578	5.23 × 10^−8^	6.91 × 10^−7^	insulin-like growth factor-binding protein 5a	*igfbp5*
Unigene0066573	−3.620	1.29 × 10^−7^	1.60 × 10^−6^	forkhead box protein H1 isoform X1	*foxh1*
Unigene0065136	−3.686	7.71 × 10^−13^	1.87 × 10^−11^	paired box protein Pax-6 isoform X1	*pax6a*
Unigene0004558	−3.928	2.86 × 10^−16^	9.70 × 10^−15^	transcription factor Sox-3 isoform X1	*sox3*
Unigene0035649	−4.122	2.35 × 10^−3^	1.29 × 10^−2^	cytochrome P450 aromatase	*cyp19a1*
Unigene0049839	−4.226	2.37 × 10^−24^	1.47 × 10^−22^	paired box protein Pax-1	*pax1*
Unigene0020541	−4.330	1.22 × 10^−36^	1.45 × 10^−34^	sperm-associated antigen 7 homolog isoform X1	*spag7*
Unigene0009593	−4.460	2.95 × 10^−46^	5.42 × 10^−44^	transcription factor SOX-11b	*sox11b*
Unigene0067666	−4.855	1.00 × 10^−40^	1.44 × 10^−38^	cytochrome P450 2J2 isoform X3	*cyp2j2*
Unigene0057503	−4.861	1.74 × 10^−9^	2.82 × 10^−8^	forkhead box protein Q1b	*foxq1b*
Unigene0057502	−5.034	3.95 × 10^−56^	1.05 × 10^−53^	insulin-like growth factor 2a	*igf2*
Unigene0036758	−5.432	3.50 × 10^−33^	3.58 × 10^−31^	transcription factor Sox-21-A	*sox21a*
Unigene0003785	−5.515	1.82 × 10^−36^	2.16 × 10^−34^	growth/differentiation factor 9	*gdf9*
Unigene0055178	−5.932	3.52 × 10^−75^	1.77 × 10^−72^	bone morphogenetic protein 15	*bmp15*
Unigene0004792	−6.643	1.53 × 10^−36^	1.81 × 10^−34^	bone morphogenetic protein 2	*bmp2*
Unigene0001965	−6.895	2.29 × 10^−89^	2.21 × 10^−86^	zygote arrest protein 1	*zar1*
Unigene0062685	−7.559	7.88 × 10^−43^	1.24 × 10^−40^	G2/mitotic-specific cyclin-B1-like isoform X1	*ccnb1*
Unigene0065315	−7.795	1.24 × 10^−33^	1.29 × 10^−31^	protein Wnt-8a ORF1 isoform X1	*wnt8a*
Unigene0063202	−9.710	7.99 × 10^−90^	7.97 × 10^−87^	zona pellucida sperm-binding protein 4-like	*zp4*
Unigene0039351	−10.013	9.72 × 10^−98^	1.51 × 10^−94^	zona pellucida sperm-binding protein 3-like	*zp3*
Unigene0011219	−10.483	7.43 × 10^−37^	9.01 × 10^−35^	zona pellucida sperm-binding protein 4-like	*zp1*
Unigene0061382	−13.657	2.08 × 10^−13^	5.35 × 10^−12^	forkhead box protein I3a	*foxi3a*

## Data Availability

The raw data supporting the conclusions of this article will be made available by the authors on request.
